# Performance characteristics of three *Brucella canis* serological assays in the United States

**DOI:** 10.3389/fvets.2025.1556965

**Published:** 2025-04-01

**Authors:** Tessa E. LeCuyer, Rebecca Franklin-Guild, Cassandra Guarino, Alexandra Fox, Kelli Maddock, Rebecca Barber, David H. Baum, Felipe Bustamante, Joshua Daniels, David M. de Avila, Dubraska Diaz-Campos, Lisa G. Glick, Jessica Haley, Sheila Heinen, Laura Leger, John Dustin Loy, Deepti Pillai, Korakrit Poonsuk, Michael M. Russell, Mithila Shukla, Erika R. Schwarz, Matthew Stempien, Deepanker Tewari, Joany C. van Balen, Stephen R. Werre, Julie T. Cecere

**Affiliations:** ^1^Department of Pathology, Microbiology, and Immunology, School of Veterinary Medicine, University of California, Davis, Davis, CA, United States; ^2^Department of Population Medicine and Diagnostic Sciences, College of Veterinary Medicine, Cornell University, Ithaca, NY, United States; ^3^Virginia Tech Animal Laboratory Services, Virginia-Maryland College of Veterinary Medicine, Virginia Tech, Blacksburg, VA, United States; ^4^North Dakota State University Veterinary Diagnostic Laboratory, North Dakota State University, Fargo, ND, United States; ^5^University of Florida Veterinary Diagnostic Laboratories, Gainesville, FL, United States; ^6^Iowa State University Veterinary Diagnostic Laboratory, Iowa State University, Ames, IA, United States; ^7^Pennsylvania Department of Agriculture, Pennsylvania Veterinary Laboratory, Harrisburg, PA, United States; ^8^Colorado State University Veterinary Diagnostic Laboratory, Fort Collins, CO, United States; ^9^Washington Animal Disease Diagnostic Laboratory, Washington State University, Pullman, WA, United States; ^10^OSU Veterinary Medical Center, Clinical Microbiology Laboratory, The Ohio State University, Columbus, OH, United States; ^11^Nebraska Veterinary Diagnostic Center, School of Veterinary Medicine and Biomedical Sciences, University of Nebraska-Lincoln, Lincoln, NE, United States; ^12^Indiana Animal Disease Diagnostic Laboratory, Purdue University, West Lafayette, IN, United States; ^13^Montana Veterinary Diagnostic Laboratory, Montana Department of Livestock, Bozeman, MT, United States; ^14^Department of Population Health Sciences, Virginia-Maryland College of Veterinary Medicine, Virginia Tech, Blacksburg, VA, United States; ^15^Department of Small Animal Clinical Sciences, Virginia-Maryland College of Veterinary Medicine, Virginia Tech, Blacksburg, VA, United States

**Keywords:** brucellosis, *Brucella canis*, serology, canine, diagnosis

## Abstract

*Brucella canis* is a zoonotic pathogen of dogs that poses diagnostic challenges. While direct detection of *B. canis* by PCR or culture is ideal, serologic diagnosis is necessary for identification of carrier animals and can support a clinical diagnosis of brucellosis. Prior to 2022, *B. canis* seroscreening in the United States was primarily performed using a commercially available rapid slide agglutination test. However, the kit was discontinued by the manufacturer in early 2022, leaving a gap in the availability of commercial *B. canis* seroassays. The goal of this study was to compare the performance of three *B. canis* serologic tests that are currently available: VMRD *Brucella ovis* ELISA, Bionote Anigen Rapid C.Brucella Ab immunochromatographic lateral flow assay, and VMRD *B. canis* indirect fluorescent antibody (IFA) assay. A panel of 56 banked serum specimens originally submitted to the Cornell University Animal Health Diagnostic Center (the study reference laboratory) for *B. canis* seroscreening was distributed to 12 testing laboratories. Each sample was run on three assays developed at the reference lab: rapid slide agglutination test with 2-mercaptoethanol (2-ME RSAT), agar gel immunodiffusion test using cytoplasmic antigen (AGID II), and Canine Brucella Multiplex. Five testing labs ran the ELISA, six ran the lateral flow, and six ran the IFA. When evaluated as a screening assay, we compared the assays to the 2ME-RSAT. The ELISA had the highest sensitivity (96.8, 95%CI 83.8–99.9) but the lowest specificity (79.3, 95%CI 57.9–92.9). The sensitivity of the lateral flow was 90.6% (95%CI 75–98%) and the IFA was 87.5% (95%CI 71–96.5). Specificity for the lateral flow was 95.8% (95%CI 78.9–99.9) and IFA was 97.5% (95%CI 67.6–97.3). When compared to AGID II and Canine Brucella Multiplex, the test assays were all highly sensitive, but specificity was <90%. Interrater reliability was highest for IFA (*Κ* = 0.92) and lowest for the lateral flow (*Κ* = 0.82). Serial testing of positive samples with a more specific test, such as AGID II, will continue to be necessary when using any of the three assays tested in this study.

## 1 Introduction

*Brucella canis*, the primary etiologic agent of canine brucellosis, is a zoonotic pathogen that is facultatively intracellular and can persist in macrophages for months to years (1, 2). Clinical signs of infection are non-specific and highly variable, ranging from testicular enlargement and abortion to ocular abnormalities and discospondylitis (1–6). This combination of inconsistent clinical signs and the intracellular, chronic persistence of the organism makes *B. canis* a diagnostic challenge (1, 3). The gold standard diagnostic method is direct detection of *B. canis* by culture, although use of PCR is increasingly described (2, 4, 5). However, direct detection is not always possible in infected animals because bacterial shedding in secretions and bacteremia may be intermittent and short-lived, with low levels of organism in circulation (1, 3).

Because of the difficulties with direct detection of *B. canis*, serologic tests are important for *B. canis* screening, as dogs remain seropositive after bacteremia has waned (2). Unfortunately, serologic tests for *B. canis* tend to have relatively low sensitivity and specificity (1, 7–9). However, they remain useful for detecting subclinical and chronic infections that may not be otherwise recognized as well as screening breeding animals (4). The serologic tests that are typically recommended for *B. canis* diagnosis and screening in the United States are the rapid slide agglutination test (RSAT), rapid slide agglutination test with 2-mercaptoethanol (2ME-RSAT), and agar gel immunodiffusion test using cytoplasmic antigen (AGID II). A commercially available RSAT (with optional 2ME step), the Zoetis D-Tec CB, was available until 2022. There is currently no commercially available source of RSAT, 2ME-RSAT, or AGID II tests or assay components in the United States, leaving a gap in diagnostic assays available for *B. canis* screening and diagnosis (5).

The goal of this study was to evaluate the performance of three methods of *B. canis* serologic testing that were available at the time of the study in the United States for use by veterinary diagnostic laboratories or as a point-of-care test by performing an interlaboratory comparison of a panel of 56 clinical serum specimens. The assays that were tested were VMRD *Brucella ovis* ELISA, Bionote Anigen Rapid C.Brucella Ab immunochromatographic lateral flow assay, and VMRD *B. canis* indirect fluorescent antibody assay (IFA). The assays were compared to the 2ME-RSAT, AGID II and Cornell University Animal Health Diagnostic Center’s (AHDC) Canine Brucella Multiplex (CBM).

## 2 Materials and methods

### 2.1 Laboratories

The participating testing laboratories were: Colorado State University Veterinary Diagnostic Laboratory (Fort Collins, CO), Indiana Animal Disease Diagnostic Laboratory – Purdue University (West Lafayette, IN), Montana Veterinary Diagnostic Laboratory (Bozeman, MT), Nebraska Veterinary Diagnostic Center (Lincoln, NE), North Dakota State University Veterinary Diagnostic Laboratory (Fargo, ND), Ohio State University Veterinary Medical Center Clinical Microbiology Laboratory (Columbus, OH), Pennsylvania Veterinary Laboratory (Harrisburg, PA), University of Florida Veterinary Hospital Clinical Microbiology Laboratory (Gainesville, FL), Virginia Tech Animal Laboratory Services (Blacksburg, VA), Washington Animal Disease Diagnostic Laboratory (Pullman, WA), Wyoming State Veterinary Laboratory (Laramie, WY). The reference laboratory was Cornell University AHDC (Ithaca, NY), which provided 2ME-RSAT, AGID II, and CBM test results. Testing laboratories volunteered to participate in the study and self-selected one or two diagnostic assays to test that were considered candidate assays for use in each respective laboratory. The ELISA was tested at five laboratories, the IFA at six laboratories, and the lateral flow assay at six laboratories. Specimens were tested September–November 2023. All laboratories had established quality assurance systems, and the staff was experienced in performing serological assays.

### 2.2 Test panel

Samples for the test panel were provided by Cornell University AHDC from banked clinical specimens and were distributed to participating laboratories. Serum samples were frozen at −20°C at Cornell University AHDC after diagnostic testing and were selected for use in the study based on the availability of sufficient volumes and prior *B. canis* serologic test results. The test panel consisted of 56 samples, which consisted of 24 specimens that were negative on 2ME-RSAT and AGID II, 7 specimens that were 2ME-RSAT positive and AGID II negative, and 25 samples that were both 2ME-RSAT and AGID II positive (20 were high positive on the CBM and 5 were low positive or equivocal on CBM). This panel was designed to have a composition appropriate for studies of assay repeatability, reproducibility, and laboratory and method comparison exercises because of the size of the panel and range of potential test results (10).

Specimens were tested on AGID II, 2ME-RSAT and CBM prior to aliquoting and shipping the specimens to the participating laboratories. Serum was shipped to the participating laboratories frozen on dry ice and stored frozen (−20°C or below) at each laboratory until testing occurred. Specimens were tested September–November 2023.

### 2.3 Dilution series

Specimens for the dilution series were prepared at Virginia Tech Animal Laboratory Services (Blacksburg, VA). Two aliquots of NVSL reagent 212-H “high positive *B. canis* Tube Agglutination Tests (TAT) control serum” (National Veterinary Services Laboratory, Ames, IA) were pooled and serial 2-fold dilutions were made using sterile 0.9% saline as the diluent. Specimens generated ranged from neat, undiluted control serum to a 1:2048 dilution. Aliquots were prepared and frozen at −20°C until shipping. Specimens were shipped to the participating laboratories on ice within 72 h of generation. The specimens were stored frozen (−20°C or below) at each laboratory until testing occurred. The dilution series specimens were run with the test panel by the same operators.

### 2.4 Seroassays

Assays performed at the Cornell University AHDC included the 2ME-RSAT, AGID II, and CBM. The 2ME-RSAT was performed using a killed whole-cell antigen derived at Cornell University from *B. canis* M- strain and stained with Rose Bengal. Patient serum was mixed with 0.2 M 2-mercaptoethanol (2-ME) as previously described (11). The AGID II was performed using a *B. canis* M-strain-derived cytoplasmic antigen and was performed as previously described (11). The CBM was performed using a multiplex fluorescent bead-based platform (Luminex) with capture reagents composed of recombinant *B. canis* proteins including BP26 and a peptide derived from Omp31 (6, 12).

Assays performed at the testing laboratories included *B. ovis* ELISA test components (VMRD, Pullman, WA), *B. canis* IFA assay (VMRD, Pullman, WA) and Rapid C.Brucella Ab immunochromatographic lateral flow assay (Bionote Anigen, Gentaur, San Jose, CA). Eleven testing laboratories ran each assay in duplicate with each run generated by a different operator. One testing laboratory ran their test assay in singlet.

The *B. canis* IFA and Bionote lateral flow were performed following manufacturer kit insert instructions. For the IFA, specimens were run at a screening dilution of 1:50. In brief, the protocol for the *B. ovis* ELISA was to dilute specimens 1:50 in serum diluting buffer and load 100 μL of diluted specimen into the antigen coated plate. Specimens were incubated for 30 min at room temperature, and then the plate was washed four times with wash buffer. Peroxidase conjugate was added, followed by another 30-min room temperature incubation and four washes. Finally, plates were developed with peroxidase substrate solution for 15 min at room temperature and then stop solution was added. Plates were read at 450 nm and optical density values were converted to S/P ratios. The cutoff for positive was S/p ≥ 0.8, which was based on canine data provided by the manufacturer.

### 2.5 Statistical analysis

Results from each laboratory were compiled by the study lead (TEL). Sensitivity and specificity of each assay were determined with respect to each of the Cornell University ADHC assays: 2ME-RSAT, AGID II, and CBM. Sensitivity and specificity calculations were based on the first result for each assay that was submitted by each laboratory. The CBM assay is semi-quantitative and therefore results were recategorized into negative (negative results) and positive (equivocal, low positive, and high positive) categories for sensitivity/specificity calculations. Exact 95% confidence intervals were calculated based on binomial probabilities. Interrater reliability was determined by Cohen’s kappa. These calculations were performed in SAS (Cary, NC). Youden’s indices were manually calculated from the sensitivity/specificity calculations. ELISA S/P ratios were compared to 2ME-RSAT results for receiver operating characteristics (ROC) analysis. The optimal ELISA cut-off was calculated by maximizing the Youden’s index with the online tool easyROC (v.1.3.1) (13).

## 3 Results

### 3.1 Serum panel

Sensitivity and specificity of each assay with respect to the reference *B. canis* seroassays 2ME-RSAT and AGID II are provided in Table 1. Interrater reliability for the test assays was highest for the IFA assay, *Κ* = 0.92 (95%CI: 0.88–0.96). The kappa scores for interrater reliability for the other assays were: ELISA *Κ* = 0.87 (95%CI: 0.82–0.93), lateral flow *Κ* = 0.82 (95%CI 0.79–0.86).

**Table 1:** Sensitivity and specificity of each test assay in the study, based on the test panel of 56 serum samples that were tested on each assay.

Test assay	Reference assay	2ME-RSAT	AGID II	CBM
Lateral flow	Sensitivity (95%CI)	90.6 (75–98)	100 (86.3–100)	100 (86.8–100)
	Specificity (95%CI)	95.8 (78.9–99.9)	83.9 (66.3–94.6)	86.7 (69.3–96.2)
	Youden’s Index	0.864	0.839	0.867
ELISA (S/P cutoff = 0.8)	Sensitivity (95%CI)	96.9 (83.8–99.9)	100 (86.3–100)	100 (86.8–100)
	Specificity (95%CI)	79.3 (57.9–92.9)	64.5 (45.4–80.8)	66.7 (47.2–82.7)
	Youden’s Index	0.762	0.645	0.667
IFA	Sensitivity (95%CI)	87.5 (71–96.5)	100 (86.3–100)	100 (86.8–100)
	Specificity (95%CI)	87.5 (67.6–97.3)	80.7(62.5–92.6)	83.3 (65.3–94.4)
	Youden’s Index	0.75	0.807	0.833

The lateral flow assay and ELISA were each tested at five labs and the IFA was tested at six labs. In the absence of a true gold standard diagnostic for the specimens in the test panel, sensitivity and specificity were calculated using each of the reference laboratory’s *Brucella canis* assays.

95%CI, 95% confidence interval; 2ME-RSAT, rapid slide agglutination test with 2-mercaptoethanol; AGID II, agar gel immunodiffusion test using cytoplasmic antigen; CBM, Canine Brucella Multiplex; IFA, indirect fluorescent antibody test.

We determined how many samples on the panel had results that were discordant with all three of the reference laboratory tests (2ME-RSAT, AGID II, and CBM) based on the results of at least one test assay in the study. The lateral flow had the highest number of serum samples that tested negative (*n* = 6) when all the reference lab tests were non-negative. The ELISA had the highest number of samples that tested positive when all the reference tests were negative (*n* = 8) (Supplemental Figure 1).

### 3.2 ELISA cutoff evaluation

Based on ROC analysis comparing the ELISA S/P ratios to reference 2ME-RSAT results, the optimal cut-off for ELISA for the data generated in this study was 0.913. Using this cutoff, the revised sensitivity of the ELISA with respect to the 2ME-RSAT was 93.1% (95%CI: 89.8–95.6) and the revised specificity was 86.7% (95%CI: 81.7–90.7). Using the revised S/P cutoff, the number of samples that tested positive on ELISA when all the reference tests were all negative was reduced to 7 samples.

### 3.3 Serial dilution series

The relative limit of detection for each assay was assessed by testing a serial dilution panel of *B. canis* TAT control serum that is available from the National Veterinary Services Laboratories (NVSL, Ames, IA) (Table 2).

**Table 2:** Two-fold serial dilutions of NVSL reagent 212-H *Brucella canis* high positive TAT control were run on each assay to compare the limit of detection of each assay on a standardized serum specimen.

Assay	Lowest dilution of 212-H that tested positive
2ME-RSAT	Neat (result = suspect)
AGID II	None (line of non-identity present)
CBM	Neat (low positive), 1:4 (equivocal)
Lateral flow	1:32–1:256
ELISA	1:16–1:32
IFA	1:32–1:128

The lowest dilution of 212-H that tested positive for each assay is listed. A range of dilutions is provided when there was variability across laboratories and/or operators in the lowest dilution detected.

## 4 Discussion

It is crucial for clinicians and veterinary diagnostic laboratories to have access to reliable diagnostic tests for *B. canis*. Because *B. canis* remains a diagnostic challenge with no single diagnostic approach that is best for every case, we performed this interlaboratory comparison to improve the understanding of the strengths and limitations of currently available *B. canis* test options in the United States. Because neither culture nor PCR status of the animals that provided serum for the test panel used in the study were available, it was not possible to determine a true “gold standard” for sensitivity and specificity calculations. Therefore, we determined the sensitivity and specificity with respect to each of the assays that is currently offered at Cornell University AHDC, the lab that has become the *B. canis* reference laboratory in the US and the only laboratory to currently offer the CBM, 2ME-RSAT, and AGID II assays for diagnostic testing.

The Bionote *B. canis* immunochromatographic lateral flow assay has not been well-characterized to date, although the manufacturer reports in the kit insert that the assay’s “overall accuracy is greater or equal to 90%” when compared to 2ME-RSAT (14). The antigen composition has not been well-described but is presumably a whole cell extract of the M (−) strain of *B. canis* based on previously published data from Bionote’s parent company, Animal Genetics, Inc., South Korea (15). Despite the lack of verified performance information for the current iteration of the test, it was adopted by many veterinarians as a point-of-care screening assay for *B. canis* upon discontinuation of the Zoetis D-tec RSAT. Several veterinary diagnostic laboratories have also started to offer this lateral flow assay after performing internal assessments of assay performance. The assay was evaluated under the name “Anigen Rapid Canine Brucella Ab Test” (manufactured by Bioeasy, South Korea) in Brazil. While the test overall had higher sensitivity than RSAT, 2ME-RSAT, and a *B. ovis* AGID, 10.4% of infected dogs (confirmed by culture and/or PCR) tested negative (8). However, of the five infected dogs in the study that tested negative, 3 had clinical signs of active brucellosis when they tested negative and likely had not seroconverted yet, as they did not test positive on RSAT or *B. ovis* AGID either. In populations in the United States in which the prevalence of *B. canis* is relatively low, such as many breeding kennels, the use of the lateral flow assay may be adequate as a screening assay but follow-up testing using another method is still required for confirmation due to the specificity of the assay when compared to the confirmatory AGID II (16, 17).

The lateral flow assay had the lowest interrater reliability of the assays tested in this study despite having the highest Youden’s index, a measure of overall diagnostic test performance. Some of the laboratories reported difficulty in reading the results for some specimens on the lateral flow assay due to the presence of non-specific colorless bands that appeared on the test kit. Although there was no color change, these areas had a slightly different color from the background of the test device and therefore could be misinterpreted as non-negative results. This, as well as the qualitative evaluation for color change in the lateral flow assay, may have led to the lower inter-rater reliability for this assay. Despite the lower agreement between test operators, the agreement for this assay could still be interpreted as “almost perfect” (18). All the test operators in this study were experienced technicians at veterinary diagnostic laboratories; it is possible that interrater agreement in a clinic environment may be lower due greater variability in experience of test operators in that setting. Due to the zoonotic implications of *B. canis* infection, it would be prudent to consider any test result that is not clearly negative as indication for confirmatory testing.

The antigen for the ELISA used in this study is *B. ovis* whole cell lipopolysaccharide extract and was designed for *B. ovis* testing. The ELISA reagents are currently available for research use only. Like *B. canis*, *B. ovis* is also a rough strain and the two species have serologic cross-reactivity (19). The ELISA components have also been tested by the manufacturer on canine serum samples to test for *B. canis* exposure. The manufacturer’s ROC analysis of ELISA S/P ratios, compared to the IFA assay, showed that a S/P cutoff of 0.8 was ideal. Our ROC analysis using the 2ME-RSAT as the reference test showed that a slightly higher S/P cutoff of 0.931 improved the specificity of the ELISA without greatly reducing the sensitivity. Follow-up testing by a more specific test such as AGID II is needed if the *B. ovis* ELISA is used as a screening test. Although ELISA results are read by an automated reader and interpreted using standard cut-off values, thereby increasing objectivity in result interpretation, this assay did not have the highest interrater reliability observed in this study.

The IFA assay uses *B. canis* strain RM666 antigen, which is the American type-strain of *B. canis* (20). We used the 1:50 screening dilution, but the manufacturer’s validation data shows a 1:100 dilution can decrease false positive reactions (21). We used a 1:50 sample dilution for this study because it was the lowest dilution regularly used by any of the labs using the IFA for diagnostic purposes. The manufacturer kit insert suggests that samples that are positive at 1:100 are “suspect” and follow-up testing with a confirmatory test such as AGID II is recommended (22).

Most *B. canis* serologic assays detect antibody against lipopolysaccharide (LPS) which may lead to cross reactions with antibody to Gram-negative bacteria (6, 23). In addition, antibodies generated against antigens on the surface of other bacteria can lead to non-specific *B. canis* serologic test results (1). The AGID II assay can avoid non-specific reactions by using cytoplasmic antigens and the CBM assay uses non-LPS antigens to avoid non-specific anti-LPS antibody binding (1, 12). Therefore, it is not surprising that the specificity of the three test assays was <90% when compared to the AGID II and CBM due to differences in the antigens across assays.

The high titer *B. canis* TAT control serum 212-H was generated as a control for the tube agglutination test, and therefore its performance has been optimized for that assay only. The reagent was obtained from a naturally infected dog and consists of lyophilized serum that contains 0.02% thimerosal as a preservative. The finding that the reference assays 2ME-RSAT and AGID II have a lower ability to detect antibody in this serum generated for use in a different test system highlights the difficulties in *B. canis* serodiagnosis. It is likely that there are antibodies to antigenic targets present in this serum sample that minimally interact with the antigens in the 2ME-RSAT and AGID II.

In our evaluation of three *B. canis* serologic assays with different test formats and antigens, each at five or six different veterinary diagnostic laboratories, we found that interrater reliability of all the assays was good. Each test assay has its own limitations and there was no single assay that had the best overall performance. The lateral flow had the lowest interrater agreement. The ELISA had the lowest specificity, but this could be mitigated in part by optimizing the S/P ratio cutoff for positive/negative results. The IFA had the lowest sensitivity when compared to the 2ME-RSAT, which has historically been used as the primary *B. canis* screening assay in the US. With knowledge of these limitations, all three of the test assays could reasonably be incorporated into a diagnostic testing algorithm for *B. canis*, but for all, a positive result on any of the tests would be an indication for follow-up testing with a more specific test (4, 5).

## Data Availability

The raw data supporting the conclusions of this article will be made available by the authors, without undue reservation.
