# Supplementary material for: Performance characteristics of three Brucella canis serological assays in the United States
Source: Front Vet Sci. 2025 Apr 1;12:1556965. doi: 10.3389/fvets.2025.1556965 (PMC11996846; doi:10.3389/fvets.2025.1556965)
Supplement: Supplementary file 1 [file Data_Sheet_1.PDF]

| Specimen | 2ME RSAT | AGID | CBM | Lateral Flow n=5 | | ELISA n=5 | | IFA n=6 | |
|---|---|---|---|---|---|---|---|---|---|
| | | | | Labs Pos | Labs Neg | Labs Pos | Labs Neg | Labs Pos | Labs Neg |
| 1 | Pos | Pos | Equivocal | 3 | 2 | 5 | 0 | 6 | 0 |
| 2 | Pos | Pos | Pos (High) | 5 | 0 | 5 | 0 | 6 | 0 |
| 3 | Pos | Pos | Equivocal | 5 | 0 | 5 | 0 | 5 | 1 |
| 4 | Pos | Pos | Pos (High) | 5 | 0 | 5 | 0 | 6 | 0 |
| 5 | Pos | Pos | Pos (High) | 4 | 1 | 5 | 0 | 6 | 0 |
| 6 | Pos | Neg | Neg | 0 | 5 | 1 | 4 | 2 | 4 |
| 7 | Pos | Pos | Pos (Low) | 3 | 2 | 5 | 0 | 6 | 0 |
| 8 | Pos | Pos | Pos (High) | 5 | 0 | 5 | 0 | 6 | 0 |
| 9 | Pos | Neg | Neg | 0 | 5 | 4 | 1 | 0 | 6 |
| 10 | Neg | Neg | Neg | 0 | 5 | 0 | 5 | 0 | 6 |
| 11 | Pos | Pos | Pos (High) | 5 | 0 | 5 | 0 | 6 | 0 |
| 12 | Pos | Neg | Neg | 0 | 5 | 2 | 3 | 0 | 6 |
| 13 | Pos | Pos | Pos (Low) | 3 | 2 | 5 | 0 | 6 | 0 |
| 14 | Pos | Neg | Neg | 3 | 2 | 5 | 0 | 6 | 0 |
| 15 | Pos | Neg | Neg | 1 | 4 | 4 | 1 | 1 | 5 |
| 16 | Neg | Neg | Neg | 2 | 3 | 5 | 0 | 6 | 0 |
| 17 | Pos | Pos | Pos (High) | 5 | 0 | 5 | 0 | 6 | 0 |
| 18 | Pos | Pos | Pos (High) | 4 | 1 | 5 | 0 | 6 | 0 |
| 19 | Neg | Neg | Neg | 0 | 5 | 0 | 5 | 0 | 6 |
| 20 | Neg | Neg | Neg | 0 | 5 | 0 | 5 | 0 | 6 |
| 21 | Neg | Neg | Neg | 0 | 5 | 1 | 4 | 0 | 6 |
| 22 | Neg | Neg | Neg | 0 | 5 | 4 | 1 | 0 | 6 |
| 23 | Neg | Neg | Neg | 0 | 5 | 0 | 5 | 0 | 6 |
| 24 | Neg | Neg | Neg | 0 | 5 | 0 | 5 | 0 | 6 |
| 25 | Neg | Neg | Neg | 0 | 5 | 0 | 5 | 0 | 6 |
| 26 | Neg | Neg | Neg | 0 | 5 | 0 | 5 | 0 | 6 |
| 27 | Neg | Neg | Neg | 0 | 5 | 5 | 0 | 0 | 6 |
| 28 | Neg | Neg | Neg | 0 | 5 | 0 | 5 | 0 | 6 |
| 29 | Neg | Neg | Neg | 0 | 5 | 0 | 5 | 0 | 6 |
| 30 | Neg | Neg | Neg | 0 | 5 | 0 | 5 | 0 | 6 |
| 31 | Neg | Neg | Neg | 0 | 5 | 3 | 2 | 1 | 5 |
| 32 | Pos | Neg | Equivocal | 3 | 2 | 5 | 0 | 6 | 0 |
| 33 | Pos | Pos | Pos (High) | 5 | 0 | 5 | 0 | 6 | 0 |
| 34 | Neg | Neg | Neg | 0 | 5 | 0 | 5 | 0 | 6 |
| 35 | Pos | Pos | Pos (High) | 5 | 0 | 5 | 0 | 6 | 0 |
| 36 | Neg | Neg | Neg | 0 | 5 | 0 | 5 | 1 | 5 |
| 37 | Pos | Pos | Pos (High) | 5 | 0 | 5 | 0 | 6 | 0 |
| 38 | Pos | Pos | Pos (Low) | 5 | 0 | 5 | 0 | 6 | 0 |
| 39 | Pos | Pos | Pos (High) | 5 | 0 | 5 | 0 | 6 | 0 |
| 40 | Neg | Neg | Neg | 0 | 5 | 0 | 5 | 1 | 5 |
| 41 | Pos | Pos | Pos (High) | 5 | 0 | 5 | 0 | 6 | 0 |
| 42 | Neg | Neg | Neg | 0 | 5 | 5 | 0 | 1 | 5 |
| 43 | Neg | Neg | Neg | 0 | 5 | 0 | 5 | 0 | 6 |
| 44 | Pos | Pos | Pos (High) | 5 | 0 | 5 | 0 | 6 | 0 |
| 45 | Pos | Pos | Pos (High) | 5 | 0 | 5 | 0 | 6 | 0 |
| 46 | Pos | Pos | Pos (High) | 5 | 0 | 5 | 0 | 6 | 0 |
| 47 | Neg | Neg | Neg | 0 | 5 | 0 | 5 | 0 | 6 |
| 48 | Neg | Neg | Neg | 0 | 5 | 1 | 4 | 0 | 6 |
| 49 | Pos | Pos | Pos (High) | 5 | 0 | 5 | 0 | 6 | 0 |
| 50 | Neg | Neg | Neg | 0 | 5 | 0 | 5 | 0 | 6 |
| 51 | Neg | Neg | Neg | 0 | 5 | 0 | 5 | 0 | 6 |
| 52 | Pos | Pos | Pos (High) | 5 | 0 | 5 | 0 | 6 | 0 |
| 53 | Pos | Pos | Pos (High) | 5 | 0 | 5 | 0 | 6 | 0 |
| 54 | Pos | Pos | Pos (High) | 5 | 0 | 5 | 0 | 6 | 0 |
| 55 | Pos | Neg | Neg | 3 | 2 | 5 | 0 | 5 | 1 |
| 56 | Pos | Pos | Pos (High) | 3 | 2 | 5 | 0 | 6 | 0 |
| Total Number of Discordant Samples for Assay | | | | 1 | 6 | 8 | 0 | 5 | 1 |

Labs pos = at least one sample at that lab was positive

gray shaded = discordant result with all of the reference lab results for that sample

**Supplemental Figure 1.** Summary of the test panel reference test results and the performance of each specimen on the test assays at each laboratory.
